# Implementation of a COVID-19 screening tool in a southern Nigerian tertiary health facility

**DOI:** 10.1371/journal.pgph.0000578

**Published:** 2022-08-26

**Authors:** Esohe O. Ogboghodo, Iriagbonse I. Osaigbovo, Darlington E. Obaseki, Micah T. N. Iduitua, Doris Asamah, Emmanuel Oduware, Benson U. Okwara

**Affiliations:** 1 Department of Public Health and Community Medicine, University of Benin Teaching Hospital, Benin City, Edo State, Nigeria; 2 Department of Medical Microbiology, University of Benin Teaching Hospital, Benin City, Edo State, Nigeria; 3 Chief Medical Director’s Office, University of Benin Teaching Hospital, Benin City, Edo State, Nigeria; 4 Accident and Emergency Department, University of Benin Teaching Hospital, Benin City, Edo State, Nigeria; 5 Department of Nursing Services, University of Benin Teaching Hospital, Benin City, Edo State, Nigeria; 6 Department of Family Medicine, University of Benin Teaching Hospital, Benin City, Edo State, Nigeria; 7 Department of Internal Medicine, University of Benin Teaching Hospital, Benin City, Edo State, Nigeria; Public Health Ontario, CANADA

## Abstract

Screening for coronavirus disease 2019 (COVID-19) in emergency rooms of health facilities during outbreaks prevents nosocomial transmission. However, effective tools adapted for use in African countries are lacking. This study appraised an indigenous screening and triage tool for COVID-19 deployed at the medical emergency room of a Nigerian tertiary facility and determined the predictors of a positive molecular diagnostic test for COVID-19. A cross-sectional study of all patients seen between May and July 2020 at the Accident and Emergency of the University of Benin Teaching Hospital was conducted. Patients with any one of the inputs- presence of COVID-19 symptoms, history of international travel, age 60 years and above, presence of comorbidities and oxygen saturation < 94%- were stratified as high-risk and subjected to molecular testing for Severe Acute Respiratory Syndrome Coronavirus 2 (SARS-CoV-2). Data was obtained from the screening record book patterned after a modified screening tool for COVID-19, deidentified and entered into IBM-SPSS version 25.0. Binary logistic regression was conducted to determine significant predictors of a positive SARS-CoV-2 test. The level of significance was set at p < 0.05. In total, 1,624 patients were screened. Mean age (standard deviation) was 53.9±18.0 years and 651 (40.1%) were 60 years and above. One or more symptoms of COVID-19 were present in 586 (36.1%) patients. Overall, 1,116 (68.7%) patients were designated high risk and tested for SARS-CoV-2, of which 359 (32.2%) were positive. Additional inputs, besides symptoms, increased COVID-19 detection by 108%. Predictors of a positive test were elderly age [AOR = 1.545 (1.127–2.116)], co-morbidity [AOR = 1.811 (1.296–2.530)] and oxygen saturation [AOR = 3.427 (2.595–4.528)]. This protocol using additional inputs such as oxygen saturation improved upon symptoms-based screening for COVID-19. Models incorporating identified predictors will be invaluable in resource limited settings.

## Introduction

Accident and emergency (A&E) departments represent the largest interface between the general public and unscheduled medical care [1]. Transmission of infections, such as measles and severe acute respiratory syndrome, to patients and health care workers is facilitated by the congested nature of most A&E departments leading to spread within hospitals [2]. During community outbreaks of highly infectious diseases such as the ongoing coronavirus disease 2019 (COVID-19) pandemic, screening at this critical interface and other facility points of entry is a *sine qua non* for the rapid identification and isolation of infected patients. The justification for screening for COVID-19 at emergency rooms is threefold. Firstly, healthcare associated transmission of COVID-19 is minimised, in-hospital outbreaks prevented and healthcare providers and patients protected. Secondly, early recognition and management of COVID-19 could prevent worsening clinical states and improve outcomes. Furthermore, excluding COVID-19 and preventing undue healthcare worker exposure maintains an active workforce required to manage the anticipated surges in patient volumes [3]. Invariably, screening ensures that health-care systems function in the face of the growing pandemic without becoming overwhelmed by COVID-19-related illness [4].

The World Health Organisation recommends screening based on the case definition for COVID-19. However, COVID-19 patients may present to healthcare facilities without the typical symptoms of fever, cough, shortness of breath, gastrointestinal disturbances, anosmia and ageusia [5, 6]. Such asymptomatic patients pose a risk to health care providers and other patients, exposing them to virus-contaminated droplet nuclei [6]. Alternative screening and triage tools applied during the pandemic have been developed in high-income countries and typically incorporate expensive and time-consuming laboratory investigations and imaging which are standard of care in these countries [1]. Implementing such tools ranges from difficult to impractical for health facilities in low- and middle-income settings where human resources, medications, laboratory testing and imaging are routinely lacking with shortages expected to worsen during times of surge [3]. With the low vaccination coverage in many African countries [7], and the emergence of highly transmissible variants [8], COVID-19 may be a recurring problem for some time to come. To sustain health service delivery during the enduring pandemic, healthcare providers in sub-Saharan Africa urgently need contextually appropriate tools to identify and triage potential COVID-19 patients. Yet, very few tools for screening have been locally developed or validated [9].

Based on early institutional experience and knowledge amassed from continuously appraising and re-appraising existing literature, staff of the University of Benin Teaching Hospital, Nigeria, designed and deployed a tool to screen for COVID-19 at the emergency room during the first wave of the pandemic [10]. This tool expanded on conventional symptoms-based screening to include oxygen saturation, age and comorbid conditions, factors which have been documented as predictors of severe illness and death from COVID-19 in many settings, including Nigeria [5, 11–16], but have not been factored into screening tools. This study aimed to appraise the outcomes of implementation of this tool and to identify predictors of COVID-19 that could be used to develop a model for low resource settings with poor access to molecular virology testing and other diagnostic facilities.

## Materials and methods

This descriptive cross-sectional study was conducted in the University of Benin Teaching Hospital (UBTH), Benin City, Edo State in the southern part of Nigeria. The UBTH is an 850-bed federal government-owned tertiary hospital offering promotive, preventive, curative and rehabilitative services in various sub-specialties [10]. It also has a designated isolation and treatment facility for COVID-19 [10] and a molecular virology laboratory with a turn-around time of 24 to 72 hours [17]. Throughout the first and subsequent waves of the COVID-19 pandemic, the facility operated a dual-track system ensuring the provision of regular health services while addressing COVID-19-related illness. Staff welfare and safety were prioritised by stratification according to risk and exemption of those at greater odds of severe COVID-19 from patient-facing tasks [18]; active surveillance for COVID-19 infection in healthcare personnel within the facility [19] and provision of personal protective equipment sourced both conventionally and through indigenous production [20]. Screening and triaging activities were instituted at every point of entry (POE) into the facility, as per WHO protocols [21], ensuring that patients were risk stratified for COVID-19 and found low risk, before accessing various services within the facility.

**Fig 1:**
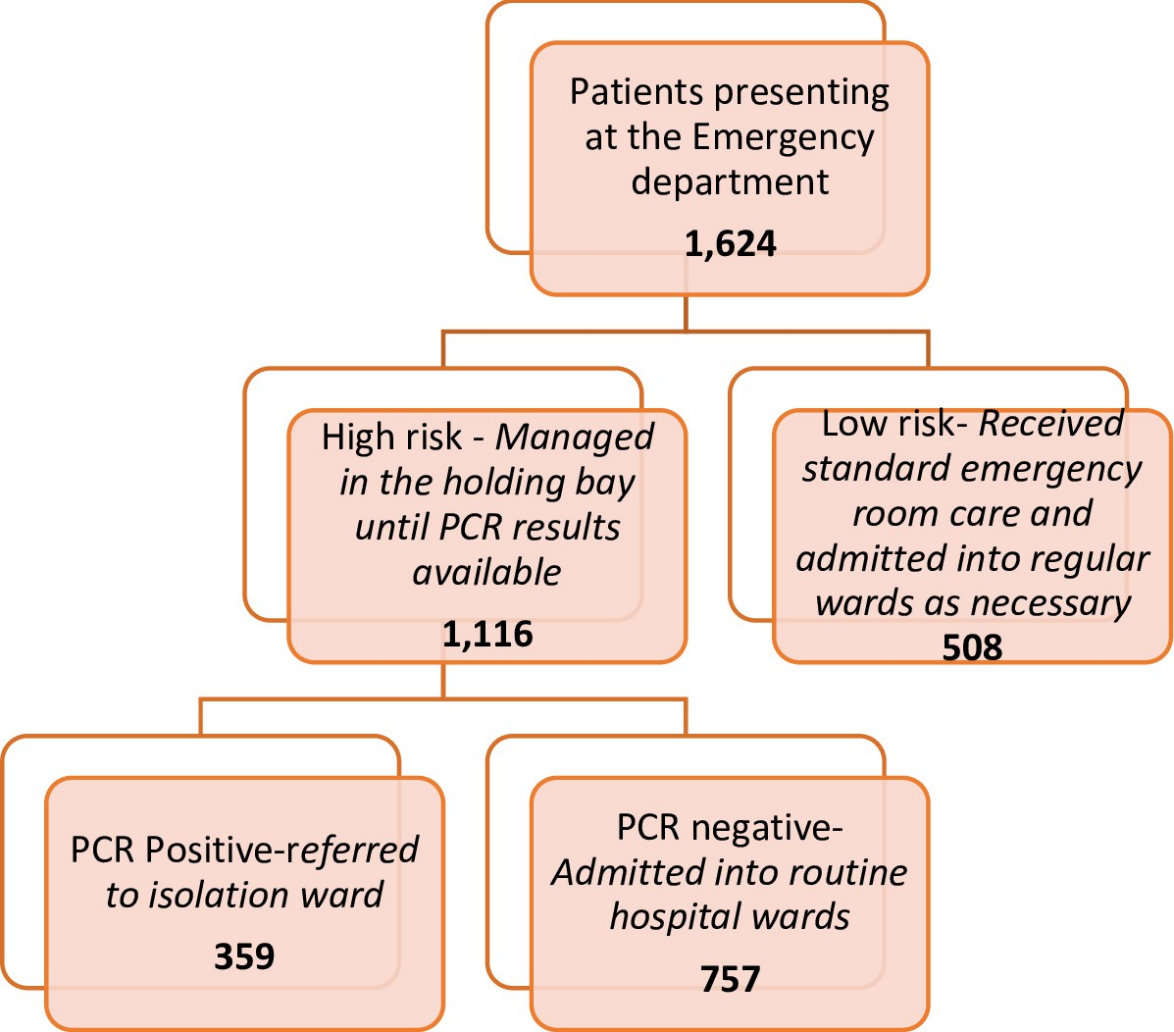
Patient flow in the A&E department.

A cross-sectional study of all patients seen at the Accident and Emergency POE between May 1^st^ and July 31^st^, 2020 was conducted. In addition to the conventional use of symptoms of COVID-19, age, presence of comorbidities and oxygen saturation were incorporated as parameters to develop a risk assessment tool which was applied to all patients presenting at the A&E. Patients aged 60 years of age and older were categorised as elderly. Those who responded ‘yes’ to any symptom of COVID-19 or additional criteria of the expanded tool (international travel history, elderly age group, presence of previously diagnosed co-morbidity including cardiovascular disease, diabetes mellitus, chronic kidney disease and cancer) and those with temperature > 37.8°C on infra-red thermometry or SPO_2_ < 94% on fingertip pulse oximetry were categorised as high risk. Nasopharyngeal (NP) and oropharyngeal (OP) swab samples were collected from high-risk patients while observing strict infection control protocols and transported to the molecular virology laboratory in viral transport medium for Severe Acute Respiratory Syndrome Coronavirus 2 (SARS-CoV-2) diagnosis by real time reverse transcriptase polymerase chain reaction (RT-PCR). Whilst awaiting the results, high-risk patients were managed as a cohort in a holding bay and staff offered care using personal protective equipment based on risk assessment of patient care activities to be performed. Patients with a positive test result were transferred to the isolation ward while those with a negative result were allowed to continue their care and access services within the facility (Fig 1).

Patient data was obtained from the POE screening record book patterned after the screening for COVID-19 and patient case records. Deidentified data for each patient was serialized, entered into IBM-SPSS version 25.0 (S1 Data) and analyzed. The statistical measures for the quantitative analysis were means with standard deviations for continuous variables and absolute numbers with percentage frequencies for categorical variables. Multivariate analysis using binary logistic regression was carried out using the ‘enter approach’ to determine significant predictors of the outcome variable, a positive SARS-COV-2 test. Age was regrouped into ages 60 years and above (elderly), and below 60 years to allow for binary logistic regression. All variables with p values ≤ 0.2 at the unadjusted level analysis were fed into the logistic regression model and analysed. The statistical measure for the analysis was the adjusted odds ratio (AOR) and 95% confidence interval (CI). The level of significance was set at p < 0.05 for all statistical associations.

To assess the improvement in COVID-19 case detection using the indigenous screening tool, percentage increase in case detection was computed by expressing the number of cases without symptoms as a percentage of the number with symptoms.

Ethical approval and a waiver of consent were obtained from the Ethics and Research Committee of University of Benin Teaching Hospital (Protocol number ADM/E22/A/VOL.VII/14830851). The hospital management also granted approval to conduct the study.

## Results

### Sociodemographic characteristics

A total of 1,624 patients were screened between May 1^st^ and July 31^st^ 2020. Their mean age (standard deviation) was 53.9 (18.0) years and 651 (40.1%) were 60 years and above. Males comprised 48.6% of patients, with a male: female ratio of 1:1.1. Two thirds 1,084 (66.7%) of the patients were married, while 198 (12.2%) were widowed (Table 1).

**Table 1:** Socio-demographic characteristics and symptom profile of patients.

Variables	Frequency (n = 1,624)	Percent
**Age Group**		
<60	973	59.9
≥ 60	651	40.1
**Mean ±SD**	**53.86 ± 18.04**	
**Sex**		
Female	834	51.4
Male	790	48.6
**Marital Status**		
Single	257	15.8
Married	1084	66.7
Divorced	85	5.2
Widowed	198	12.2
**Presence of symptoms (n = 1624)**		
Yes	586	36.1
No	1038	63.9
**If yes, what symptoms (n = 586)** ^**a**^		
Fever	318	54.3
Difficulty with breathing	249	42.5
Cough	233	39.8
Myalgia	221	37.7
Vomiting	68	11.6
Sore-throat	49	8.4
Runny-nose	44	7.5
Nausea	32	5.5
Anosmia	29	4.9
Ageusia	18	3.1

^a^Multiple responses

On screening, 586 (31.6%) patients reported one or more symptoms of COVID-19: 318 (54.3%) had fever, 249 (42.5%) presented with difficulty with breathing and 233 (39.8%) presented with cough. Five hundred and fourteen (87.7%) of the symptomatic patients had one or more major symptoms (fever, cough and difficulty in breathing). Less common symptoms of COVID-19 seen at the POE were nausea in 32 (5.5%), anosmia in 29 (4.9%) and ageusia in 18 (3.1%; Table 1).

### Risk stratification

Risk assessment of the patients using presence of symptoms and additional criteria (international travel history, elderly age group, presence of co-morbidity and SPO_2_ < 94%) is shown in Table 2. Presence of symptoms automatically qualified a patient as high-risk. The presence of any of the additional criteria was likewise regarded as high risk. Overall, 1,116 (68.7%) patients screened at the A&E were stratified as high risk and were tested for COVID-19. Of these, 359 (32.2%) were positive.

**Table 2:** Risk assessment for COVID-19 at A&E point of entry and test result.

Variable	Frequency (n = 1,624)	Percent
**Elderly Age Group**		
No	973	59.9
Yes	651	40.1
**Presence of Comorbidities** *		
No	898	55.3
Yes	726	44.7
**Symptoms at Presentation**		
No	1,038	63.9
Yes	586	36.1
**Oxygen saturation**		
Normal	1,046	64.4
Hypoxia	578	35.6
**Travel history**		
No	1,584	97.5
Yes	40	2.5
**Risk assessment**		
Low Risk	508	31.3
High Risk	1,116	68.7
**Positive Covid-19 Test Result (n = 359)**		
Symptomatic	172	47.9
Non symptomatic	187	52.1

*Comorbidities were cardiovascular disease, diabetes mellitus, chronic kidney disease and cancer.

### Improvements in case detection ascribable to the indigenous screening tool

The total number of COVID-19 cases detected among high-risk patients was 359, of which 172 (47.9%) had one or more symptoms. The use of the indigenous tool resulted in the detection of another 187 cases, a 108.7% increase in the number based on symptoms alone.

### Predictors of a positive RT-PCR for SARS-CoV-2 result

At bivariate level analysis, age group (p<0.001), sex (p = 0.053), symptoms at presentation (p = 0.034), presence of co-morbidities (p<0.001) and SPO_2_ (p<0.001) were selected to feed into the multivariable regression model while travel history was excluded (p = 0.531). At the multivariable level, elderly age group, presence of co-morbidities and hypoxia were the significant predictors of a positive result (Table 3). Patients who were elderly were 1.5 times more likely to have a positive RT-PCR for SARS-CoV-2, compared to persons aged less than 60 (AOR = 1.545, CI = 1.127–2.116). Also, patients who had co-morbidities were 1.8 times more likely to have a positive RT-PCR for SARS-CoV-2, compared to persons who did not have any co-morbidity (AOR = 1.811, CI = 1.296–2.530). Patients who were hypoxic at presentation were 3.4 times more likely to have a positive RT-PCR for SARS-CoV-2 compared to persons with a normal SPO_2_ level (AOR = 3.427, CI = 2.595–4.528). Although males were 1.3 times more likely to have a positive test, sex was not a significant predictor after controlling for variables at the multivariable level (AOR = 1.262, CI = 0.966–1.648).

**Table 3:** Unadjusted and adjusted logistic regression model for determinants of a positive RT-PCR for SARS-CoV-2 result.

Predictors	SARS-CoV-2 Prevalence (%)	Unadjusted OR (95% CI)	p-value	Adjusted OR (95% CI)	p-value
**Age group (years)**					
≥ 60	24.5	1.858 (1.427–2.420)	<0.001	1.545 (1.127–2.116)	0.007
< 60	37.6	1		1	
**Sex**					
Male	34.8	0.779 (0.605–1.003)	0.053	1.262 (0.966–1.648)	0.087
Female	29.3	1		1	
**Symptoms at presentation**					
Yes	29.4	0.762 (0.592–0.980)	0.034	0.394 (0.889–0.679)	0.394
No	35.3	1		1	
**Presence of Co-morbidities** *					
Yes	37.5	2.087 (1.574–2.766)	<0.001	1.811 (1.296–2.530)	0.001
No	22.3	1		1	
**SPO**_**2**_					
Hypoxia	44.1	3.295 (2.516–4.314)	<0.001	3.427 (2.595–4.528)	<0.001
Normal	19.3	1		1	

* Comorbidities were cardiovascular disease, diabetes mellitus, chronic kidney disease and cancer.

## Discussion

The COVID-19 pandemic is one of the most formidable challenges ever faced by modern healthcare. Many African countries, including Nigeria, have now encountered multiple waves of the pandemic [22]. Amidst the recurring nature of the crisis lies an opportunity to plan for future waves by relying on lessons learnt in the not-so-distant past. This cross-sectional study of data accrued from the emergency department of our hospital during the first wave quantifies the advantage of an enhanced screening tool over symptoms-based screening for COVID-19 case detection. By inputting age, oxygen saturation and presence of co-morbidities into our tool, we were able to identify additional cases of COVID-19 that would otherwise have been missed because they did not have typical symptoms of the disease. This translated to an over 100% increase in the number of cases detected.

The absence of commonplace symptoms in a large number of infected persons invalidates the use of symptoms alone for predicting COVID-19 infection [23]. In addition, symptom-based screening is rendered non-specific by the high prevalence of HIV, malaria, respiratory disease and other communicable diseases with similar presentations in tropical regions. On the whole, symptoms were not a significant predictor in the current study. Similarly, studies conducted in Lagos, Nigeria [24], Ghana [25] and the United States [26] prove that the predictive ability of symptomatology for COVID‑19 is extremely weak. Considered individually, anosmia and ageusia are the only symptoms which have been found to be highly predictive of COVID-19 [27]. However, these were relatively rare, affecting less than one in twenty cases in our cohort. Findings from a meta-analysis reported that frequency of taste or smell alteration was higher in studies that recruited outpatients than those that only recruited hospitalised patients [27] and this may explain the rarity in our study. Our experience at the UBTH also points to taste and smell alteration as symptoms occurring towards the tail end of COVID-19 illness, often signaling recovery.

This study identified hypoxaemia as the strongest predictor of a positive COVID-19 test among high-risk patients. Hypoxaemia occurring in the absence of dyspnoea, the so-called silent hypoxia, has been described in patients with early stage COVID-19 and is established both internationally and locally, as a predictor of disease severity [28–31]. The pathophysiological mechanisms are not well understood but COVID-19 patients with “silent hypoxia” often have radiological features of pneumonia and may go ahead to develop severe disease requiring assisted ventilation and intubation [28]. Silent hypoxia can be detected with the aid of pulse oximetry. Despite the relatively low cost of fingertip pulse oximeters, emergency rooms in many low resource settings lack the device. A national survey of hospital readiness during the pandemic showed that only 15% of 20 Nigerian hospitals assessed had an adequate supply of this critical instrument. Another 65% had pulse oximeters but the quantities were inadequate and 20% had none [32]. In addition, a recent review of available studies involving adults also demonstrated a dire lack of access to oxygen delivery systems across sub-Saharan Africa with most facilities ill-equipped to identify adult patients with hypoxemia [33]. WHO guidelines on the clinical use of oxygen and technical specifications for oxygen equipment are available but emphasis is on children with specifications for adults conspicuously lacking [34]. Likewise, available data on quality improvement efforts at increasing oxygen access and pulse oximetry in Nigeria have hitherto focused on children, which is understandable given the high mortality from pneumonia for children under five years [35, 36]. Ironically, the paediatric population has been relatively spared during the present pandemic [37]. Our findings provide support for the increasingly popular notion that pulse oximetry become routine practice not only in paediatric but also adult emergency rooms, with oxygen saturation adopted as the ‘fifth sign’ in basic vital sign documentation [38].

Although the indigenous tool aided the prioritization of patients for testing, only about a third of these eventually tested positive. This is an inherent characteristic of screening, which prioritises high sensitivity over specificity so that all cases can be identified. However, provision of care to patients suspected of having COVID-19 requires specialized precautions, including dedicated diagnostic and treatment spaces, trained healthcare personnel and large quantities of personal protective equipment to ensure infection control while molecular testing results are being awaited [39]. Cohorting all patients under investigation may also inadvertently place those who turn out to be uninfected at excess risk for healthcare-related exposure to SARS-CoV-2 [39]. Nevertheless, for our facility, a federal government owned tertiary institution with a molecular laboratory onsite, we are convinced that the benefits of limiting nosocomial transmission outweighed the inconvenience and costs of managing suspected cases. Facilities with less funding, understaffing, limited physical space for isolation and remote access to molecular diagnosis, on the other hand, may become overwhelmed. The use of well validated antigen based rapid diagnostic tests may further reduce molecular testing needs while helping to decongest holding bays for suspect cases. Implementation and cost-benefit analysis studies are needed to assess the usefulness of our screening strategy alone and in combination with rapid antigen testing.

This study is one of few to present primary data on the subject of screening and triage in low resource settings in Africa. However, it has some limitations, one of which is that the accuracy of our tool cannot be ascertained since individuals stratified as low-risk were not tested. The study relies on data accrued during the first wave of the pandemic and it is not known how the emergence of SARS-COV-2 variants might affect its applicability. Although PCR is considered the gold standard, NP and OP swabs may give false negative results and COVID-19 has been reported using computed tomography in people who had a negative PCR test from upper respiratory samples [40]. An underestimated prevalence of COVID-19 among the high-risk group is, thus, another possible limitation of our study. As stated previously, this tool’s utility is limited by its high false positive rate as it might inadvertently put those who are actually uninfected at risk of contracting the disease while awaiting results of the PCR test. To minimize this risk, patients in the holding area were masked and a distance of three metres was maintained between beds. Appropriate protective equipment was also used by staff performing patient care activities according to risk assessment. Finally, our screening tool was used in an adult emergency room and may not be appropriate for paediatric populations. Ultimately, the feasibility of implementing this tool could be dictated by other factors such as the dominant SARS-CoV-2 variant, patient volume and laboratory capacity among others.

In conclusion, we have appraised the usefulness of a robust screening tool which incorporated parameters obtainable from history taking, and easy-to-use, relatively affordable instruments like a fingertip pulse oximeter thus enabling feasibility in low- and middle-income African settings. Implementation of this tool with well validated rapid diagnostic tests will likely reduce molecular testing needs while limiting nosocomial transmission of SARS-CoV-2. We also identified the predictors of a positive COVID-19 test in high-risk patients which would be invaluable in developing and validating a model that can be applied during future COVID-19 outbreak waves in settings with limited access to molecular testing facilities.

## Supporting information

S1 Data SPSS data sheet. Data underlying conduct of this study.
(SAV)
