# Correction: Implementation of a COVID-19 screening tool in a southern Nigerian tertiary health facility

**DOI:** 10.1371/journal.pgph.0002299

**Published:** 2023-08-08

**Authors:** Esohe O. Ogboghodo, Iriagbonse I. Osaigbovo, Darlington E. Obaseki, Micah T. N. Iduitua, Doris Asamah, Emmanuel Oduware, Benson U. Okwara

## Notice of republication

In light of an issue identified post-publication, this article was republished on September 7, 2022, to replace S1 Dataset and remove information that was not relevant to the article. Please download this article again to view the correct version.
